# Virtual bronchoscopic navigation without X-ray fluoroscopy to diagnose peripheral pulmonary lesions: a randomized trial

**DOI:** 10.1186/s12890-017-0531-2

**Published:** 2017-12-11

**Authors:** Fumihiro Asano, Takashi Ishida, Naofumi Shinagawa, Noriaki Sukoh, Masaki Anzai, Kenya Kanazawa, Akifumi Tsuzuku, Satoshi Morita

**Affiliations:** 1 Department of Pulmonary Medicine, Gifu Prefectural General Medical Center, 4-6-1, Noishiki, Gifu, 500-8717 Japan; 2 Department of Pulmonary Medicine, Fukushima Medical University, 1, Hikariga-oka, Fukushima City, 960-1295 Japan; 3 First Department of Medicine, Hokkaido University School of Medicine, North 15, West 7, Kita-ku, Sapporo, 060-8638 Japan; 4 Department of Pulmonary Diseases, National Hospital Organization Hokkaido Cancer Center, 4-2-3-54, Kikusui, Shiroishi-ku, Sapporo, 003-0804 Japan; 5 Department of Pulmonary Medicine, Fukui University School of Medicine, 23-3, Matsuoka-Shimoaizuki, Eiheiji-cho, Yoshida-gun, Fukui, 910-1193 Japan; 6 Department of Biomedical Statistics and Bioinformatics, Kyoto University Graduate School of Medicine, 54, Kawahara-cho, Shogoin, Sakyo-ku, Kyoto, 606-8507 Japan

**Keywords:** Bronchoscopy, Endobronchial ultrasonography, Guide sheath, Lung cancer, Peripheral pulmonary lesion, Transbronchial biopsy, Virtual bronchoscopic navigation, X-ray fluoroscopy

## Abstract

**Background:**

Transbronchial biopsy for peripheral pulmonary lesions is generally performed under X-ray fluoroscopy. Virtual bronchoscopic navigation (VBN) is a method in which virtual images of the bronchial route to the lesion are produced based on CT images obtained before VBN, and the bronchoscope is guided using these virtual images, improving the diagnostic yield of peripheral pulmonary lesions. VBN has the possibility of eliminating the need for X-ray fluoroscopy in the bronchoscopic diagnosis of peripheral lesions. To determine whether VBN can be a substitute for X-ray fluoroscopy, a randomized multicenter trial (non-inferiority trial) was performed in VBN and X-ray fluoroscopy (XRF) -assisted groups.

**Methods:**

The non-inferiority margin in the VBN-assisted group compared with the XRF-assisted group was set at 15%. The subjects consisted of 140 patients with peripheral pulmonary lesions with a mean diameter > 3 cm. In the VBN-assisted group, the bronchoscope was guided to the lesion using a VBN system without X-ray fluoroscopy. In the XRF-assisted group, the same bronchoscope was guided to the lesion under X-ray fluoroscopy. Subsequently, in both groups, the lesion was visualized using endobronchial ultrasonography with a guide sheath (EBUS/GS), and biopsy was performed. In this serial procedure, X-ray fluoroscopy was not used in the VBNA group.

**Results:**

The subjects of analysis consisted of 129 patients. The diagnostic yield was 76.9% (50/65) in the VBN-assisted group and 85.9% (55/64) in the XRF-assisted group. The difference in the diagnostic yield between the two groups was -9.0% (95% confidence interval: -22.3% ~ 4.3%). The non-inferiority of the VBN-assisted group could not be confirmed. The rate of visualizing lesions by EBUS was 95.4% (62/65) in the VBN-assisted group and 96.9% (62/64) in the XRF-assisted group, being high in both groups.

**Conclusions:**

On EBUS/GS, a bronchoscope and biopsy instruments may be guided to the lesions using VBN without X-ray fluoroscopy, but X-ray fluoroscopy is necessary to improve the accuracy of sample collection from lesions. During transbronchial biopsy for peripheral pulmonary lesions, VBN cannot be a substitute for X-ray fluoroscopy.

**Trial registration:**

UMIN-CTR (UMIN000001710); registered 16 February 2009.

## Background

With the recent spread of CT screening, pulmonary peripheral lesions have been increasingly detected [1]. The previously reported diagnostic yield employing transbronchial biopsy for pulmonary peripheral lesions widely varies from 36 to 86% [2–5]. This may be partly because pulmonary peripheral lesions and the arrival of the biopsy forceps or brush for cytological diagnosis at the lesion cannot be confirmed using X-ray fluoroscopy alone. To overcome this problem, transbronchial biopsy after confirming the lesion using CT fluoroscopy [6] or endobronchial ultrasonography (EBUS) [7] has been used. In addition, EBUS with a guide sheath (EBUS/GS) [8] was developed and has produced favorable results in recent years [9, 10]. However, EBUS/GS is originally a method to confirm the arrival of the biopsy instruments at the lesion. For the guidance of a bronchoscope and biopsy instruments to the lesion before the confirmation of their arrival, X-ray fluoroscopy is still necessary. X-ray fluoroscopy during bronchoscopy involves X-ray exposure of the patient and operator, and requires X-ray fluoroscopy equipment and a special room [11]. To overcome these problems, Yoshikawa et al. reported the results of EBUS/GS without X-ray fluoroscopy [12], but this method is not commonly performed.

In addition to EBUS/GS, several guided-bronchoscopy technologies have recently been developed to improve the yield of transbronchial biopsy for diagnosing peripheral lesions [13]. We have proposed virtual bronchoscopic navigation (VBN) that uses virtual images of the route to the lesion for the guidance of a bronchoscope [14, 15]. Electromagnetic navigation (EMN) is a method to guide a guide sheath and biopsy instrument to peripheral lesions utilizing electromagnetic position-sensor information, and it is performed without X-ray fluoroscopy in many cases [16]. On the other hand, VBN is a method to guide a bronchoscope (normally a thin endoscope) close to the lesion by making the bronchial route on virtual images consistent with actual bronchoscopic images without using a position sensor, being different from EMN. VBN systems have been developed and become commercially available. There have already been some studies on VBN [12, 17–27]. A randomized study on transbronchial biopsy when used together with EBUS/GS and X-ray fluoroscopy showed that VBN increased the diagnostic yield and reduced the examination time [25]. VBN is advantageous in that the diagnostic yield is high and it requires no expensive disposable position sensor used for EMN, but X-ray fluoroscopy has been considered necessary to confirm arrival of the biopsy instrument at the lesion because no position sensor is used. VBN determines the route to the lesion based on CT images obtained before VBN, and therefore, does not require X-ray fluoroscopy and can guide the bronchoscope to the target. If lesions involving bronchi on CT images are selected, and EBUS/GS is guided by VBN, diagnosis may be possible without using X-ray fluoroscopy. Since the diagnostic yield of examination using VBN is as high as that using EMN [13, 28], if the bronchoscopic diagnosis of peripheral lesions is possible using VBN without X-ray fluoroscopy, similarly to that using EMN, it would eliminate intraoperative X-ray exposure, being markedly advantageous.

We conducted a multi-center, randomized clinical trial to confirm that the diagnostic yield does not decrease in a group using VBN instead of X-ray fluoroscopy (VBN-assisted group, VBNA group) compared with a group using X-ray fluoroscopy (XRF-assisted group, XRFA group) in the bronchoscopic diagnosis of pulmonary peripheral lesions when used together with EBUS/GS (non-inferiority trial).

## Methods

### Study subjects

Eligible subjects were adults (≥ 20 years) with peripheral pulmonary lesions (mean diameter, > 30 mm calculated from axial CT images) suspected to be cancer but were not pathologically confirmed [25]. Peripheral pulmonary lesions were defined as lesions surrounded by normal lung parenchyma and were unlikely to be visualized by bronchoscopy. Yoshikawa et al. reported the usefulness of EBUS/GS without X-ray fluoroscopy for diagnosing large lesions and lesions involving the bronchus [12]. Since the rate of identifying large lesions involving the bronchus is high, the target of this study was set at 30-mm or larger lesions.

Exclusion criteria included evidence of endobronchial disease as observed using an bronchoscope and conditions for which transbronchial biopsy is not indicated as previously described [25]. In addition, lesions mainly observed as ground glass opacity (GGO) on CT were included in the exclusion criteria because these lesions may be difficult to definitely diagnose pathologically even though the specimen is collected from the lesion.

### Study design

This randomized clinical trial was conducted at 5 Japanese medical centers between February 2009 and November 2011. Sample size was calculated on the basis of the primary endpoint. Based on our experience, when the diagnostic yield in the XRFA and VBNA groups was expected to be 90%, and the non-inferiority margin in the VBNA group compared with the XRFA group was regarded as 15%, the number of patients necessary for analysis with an α error of 5% (two-sided) and a power of 80% was estimated to be 63 in each group. Considering that some patients would be excluded from analysis, the planned number of patients was determined to be 70 in each group (140 in total).

### Randomization

Eligible patients were randomly assigned to the VBNA or XRFA groups. Because the diagnostic yield of bronchoscopy has been shown to be associated with physician skill [29], randomization was based on bronchoscopists used the permuted block randomization method to ensure that this factor were balanced in the study arms.

### Intervention

Scan data from multidetector chest CT (16- or 64-row; slice width, 0.5-1.5 mm, recommendation, 0.5 mm) were acquired from all patients before bronchoscopy. The same CT conditions were used at each institution. Individual CT datasets from the VBNA group were transferred to a workstation on which VBN software (Bf-NAVI; Cybernet Systems, Tokyo, Japan) automatically created VB images of the target bronchus most likely leading to the pulmonary peripheral lesion [25]. Bronchoscopy was performed under moderate sedation and local anesthesia. In the VBNA group, a thin bronchoscope (type P260F; outer diameter, 4.0 mm; working channel diameter, 2.0 mm; Olympus Medical Systems) was navigated to the target bronchus using the VBN system without X-ray fluoroscopy. In the XRFA group, the same type of thin bronchoscope was advanced to the target bronchus without VBN support and with reference only to CT axial images under X-ray fluoroscopy. After the bronchoscope was advanced to the target bronchus near the lesion, a 20-MHz mechanical radial-type endobronchial ultrasonic (EBUS) probe (external diameter, 1.4 mm; UM-S20-17R; Olympus Medical Systems) with a guide sheath (external diameter, 1.95 mm, K-201; Olympus Medical Systems) was inserted through a working channel. The EBUS probe was withdrawn as soon as the lesion was visualized. Pathological samples were collected from the same lesion site as verified by EBUS using forceps and/or a brush introduced into the guide sheath [19, 24]. The area around the bronchial target was washed with 20 mL of saline. Pathologists, blinded to the results of the randomization, processed and evaluated all the specimens using standard procedures.The presence of bacteria was assessed in some portions of brush smears and/or lavage.

In this serial procedure (guidance of the bronchoscope, confirmation of the lesion using EBUS/GS and biopsy) for the bronchoscopic diagnosis of peripheral pulmonary lesions, X-ray fluoroscopy was not used in the VBNA group. When the lesion could not be visualized by EBUS/GS, the diagnosis was considered to be impossible, and examination was continued using X-ray fluoroscopy. In the XRFA group, X-ray fluoroscopy was used only in the following cases: 1) Until the confirmation of the lesion by EBUS/GS, X-ray fluoroscopy was used to confirm positional relationships among the bronchoscope, EBUS probe with a guide sheath, curette type inductor, and the lesion. When the lesion could not be visualized after insertion of the EBUS probe alone, the lesion was visualized after the guide sheath had been advanced to the lesion using a curette-type inductor under the guidance of X-ray fluoroscopy. 2) After confirming the lesion by EBUS, X-ray fluoroscopy was used to confirm the absence of a shift of the biopsy instrument to the lesion in each biopsy procedure.

### Study follow-up

If a lesion was not diagnosed by bronchoscopy, we recommended that the patient consider undergoing other diagnostic procedures, including repeated bronchoscopy, CT-guided transthoracic needle aspiration, or surgical intervention. If a patient with an undiagnosed lesion refused further intervention, follow-up for 2 years was considered the second best strategy. Final diagnoses were established by evaluating pathological evidence from biopsies, including bronchoscopic or surgical procedures, microbiological analysis, or clinical follow-up.

### Outcomes

The primary endpoint was the diagnostic yield, as a measure of the non-inferiority of VBNA group compared with the XRFA group. In the diagnosis of benign diseases, inconclusive histological results such as nonspecific fibrosis or inflammation were analyzed as nondiagnostic [30]. The diagnostic yield of bronchoscopy was analyzed in subgroups classified according to the following items: lesion size, lung lobe containing the lesion, whether the lesion was detected by frontal X-P, presence or absence of a bronchus sign [31], lesion location, whether the lesion was visualized by EBUS, and final diagnosis. The location was classified into central, intermediate, and peripheral thirds according to the distance from the hilum based on the study of Baaklini et al. [2]. Each item was evaluated by 2 or more physicians before bronchoscopy.

As for the secondary endpoint, the following items were evaluated: bronchial generation of VB images, endoscopically inserted bronchial generation (all subsegmental bronchi were regarded as 3rd-generation bronchi, and bronchial generation was calculated by adding the number of further branchings), agreement between VB images and actual bronchi, confirmation of the lesion by EBUS, number of samplings by biopsy and brushing, total examination time, examination time before sample collection, total X-ray fluoroscopy time, and X-ray fluoroscopy time before sample collection [25].

The safety endpoints of interest included hemorrhage, pneumothorax, hypoxemia, lidocaine intoxication, arrhythmia, pneumonia, and other serious adverse events. A retrieved blood loss of >50 mL mixed with or without saline lavage was defined as significant.

### Statistical analyses

Noninferiority of the VBN without X-ray fluoroscopy (VBNA group) was to be concluded if the lower bound of the 95% confidence interval (CI) for the difference in the diagnostic yields exceeded the predetermined noninferiority bound of −15%. Except for the noninferiority analysis of the primary endpoint, categorical variables were analyzed using the Pearson χ2 test or the Fisher exact test; continuous variables were assessed for normality in distribution and the Mann-Whitney U test/description as medians was used. All *P*-values were two-sided. A P-value of <0.05 was considered to indicate a statistically significant difference. A Bonferroni correction was not used to deal with the multiplicity problem in this study. All data were statistically analyzed using IBM SPSS Statistics, version 19 (SPSS Inc., Chicago, IL).

## Results

**Fig. 1:**
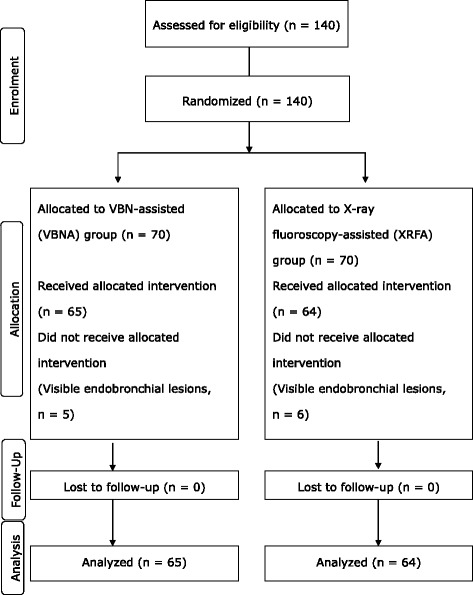
CONSORT flow diagram. VBN, virtual bronchoscopic navigation

The phases of the clinical trial are shown in Fig. 1. The subjects for the analysis consisted of 129 patients [VBNA group (*n* = 65) and XRFA group (*n* = 64)]. There were no significant differences in the baseline characteristics between both groups in the randomized population (Table 1). Among the bronchoscopically undiagnosed patients, 4 patients (16.6%) of 24 underwent video-assisted thoracoscopy or surgery, and the conditions of 13 patients (54.2%) were diagnosed by CT-guided transthoracic needle aspiration or repeated bronchoscopy. Seven patients (29.2%) who refused further intervention were followed up for 2 years.

**Table 1:** Baseline characteristics and final diagnoses in a randomized population

	VBNA group (*n* = 65)	XRFA group (*n* = 64)	*P*-value
Age (y, median; range)	71 [48–86]	70.5 [53–84]	0.794
Male, n (%)	46 (70.8)	52 (81.3)	0.217
Lesion size (mm, median; range)	37.5 [30–97]	39.0 [30–84]	0.823
≤ 50 mm, n (%)	49 (75.4)	55 (85.9)	0.181
Lesion location			0.313
Rt. upper lobe, n (%)	19 (29.2)	14 (21.9)	
Rt. middle lobe, n (%)	5 (7.7)	2 (3.1)	
Rt. lower lobe, n (%)	13 (20.0)	19 (29.7)	
Lt. upper lobe, n (%)	21 (32.3)	17 (26.6)	
Lt. lower lobe, n (%)	7 (10.8)	12 (18.8)	
Final diagnosis			0.090
Primary lung cancer, n (%)	56 (86.2)	60 (93.8)	
Other malignant diseases, n (%)	3 (4.6)	2 (3.1)	
Non-malignant disease, n (%)	6 (9.2)	2 (3.1)	
Frontal X-ray film			0.493
Invisible	2 (3.1)	3 (4.7)	
Visible	63 (96.9)	61 (95.3)	
Bronchus sign			0.119
Absent	0 (0)	3 (4.7)	
Present	65 (100)	61 (95.3)	
Location			0.345
Central	7 (10.8)	4 (6.3)	
Intermediate	11 (16.9)	17 (26.6)	
Peripheral	47 (72.3)	43 (67.2)	

The diagnosis yield was 76.9% (50/65) in the VBNA group and 85.9% (55/64) in the XRFA group. The difference in the diagnostic yield between the two groups was -9.0% (95% confidence interval: -22.3% ~ 4.3%). The non-inferiority of the VBNA group could not be confirmed. There was no significant difference in the diagnostic yield between the groups in the subgroup analysis (Table 2).

**Table 2:** Diagnostic yield according to each parameter

	VBNA	XRFA	*P*- value	*Odds ratio*	*95% Confidence* *interval*	
Total	50/65 (76.9)	55/64 (85.9)	0.258	1.8	0.7	4.6
Forceps	48/65 (73.8)	54/64 (84.4)	0.142	1.9	0.8	4.6
Brush	40/65 (61.5)	47/64 (73.4)	0.149	1.7	0.8	3.6
Lesion size						
≤ 50 mm	36/49 (73.5)	46/55 (83.6)	0.236	1.8	0.7	4.8
> 50 mm	14/16 (87.5)	9/9 (100)	0.400	1.1	1.0	1.4
Lobe						
Right upper	15/19 (78.9)	13/14 (92.9)	0.278	3.5	0.3	35.1
Right middle	4/5 (80.0)	2/2 (100)	0.714	N/A		
Right lower	12/13 (92.3)	14/19 (73.7)	0.197	0.2	0.02	2.3
Left upper	14/21 (66.7)	15/17 (88.2)	0.120	3.8	0.7	21.2
Left lower	5/7 (71.4)	11/12 (91.7)	0.296	4.4	0.3	60.6
Frontal X-ray film						
Invisible	1/2 (50.0)	3/3 (100)	0.400	N/A		
Visible	49/63 (77.8)	52/61 (85.2)	0.357	1.7	0.7	4.2
Bronchus sign						
Absent	0/0 (0)	3/3 (100)	N/A	N/A		
Present	50/65 (76.9)	52/61 (85.2)	0.263	1.7	0.7	4.3
Location						
Central	5/7 (71.4)	3/4 (75.0)	0.721	1.2	0.07	19.6
Intermediate	8/11 (72.7)	15/17 (88.2)	0.290	2.8	0.4	20.5
Peripheral	37/47 (78.7)	37/43 (86.0)	0.418	1.7	0.5	5.1
EBUS						
Visualized	50/62 (80.6)	55/62 (88.7)	0.319	1.9	0.7	5.2
Not visualized	0/3 (0)	0/2 (0)				
Final diagnosis						
Primary lung cancer	43/56 (76.8)	53/60 (88.3)	0.140	2.3	0.8	6.2
Metastatic lung tumor	2/3 (66.7)	1/2 (50.0)	0.700	0.5	0.01	19.6
Benign	5/6 (83.3)	1/2 (50.0)	0.464	0.2	0.01	6.6

Data are shown as numbers of lesions/total lesions (%)

The median generation of virtual bronchial images constructed based on data from the VBNA group was 4 (Table 3). The VBN system thus allowed the insertion of the thin bronchoscope into significantly further generations of the bronchi. A total of 93.8% of virtual images were in agreement with the actual images for the shape of each bronchial bifurcation on the route. There was no difference in the confirmation rate of the lesion by EBUS in both groups. The examination time was shorter in the VBNA than XRFA group, but the difference was not significant.

**Table 3:** Bronchoscopic outcomes in the analysed population

	VBNA	XRFA	*P*-value
Bronchial generation of virtual bronchoscopic images (n, median) [range]	4 [2-7]	N/A	N/A
Endoscopically inserted bronchial generation (n, median) [range]	4 [1-6]	3 [1-6]	0.025
Agreement between virtual bronchoscopic images and actual bronchi, n (%)	61/65 (93.8)	N/A	N/A
Confirmation of lesion by EBUS, n (%)	62/65 (95.4)	62/64 (96.9)	0.507
Sampling by biopsy, (n, median) [range]	5 [1-7]	5 [0-12]	0.434
Sampling by brushing (n, median) [range]	2 [0-4]	2 [1-5]	0.983
Duration			
Total examination (min, median) [range]	16.6 [7.6-36.5]	18.5 [8.3-55.4]	0.081
Examination before sample collection, (min, median) [range]	5.9 [2.2-18.2]	6.2 [2.0-33.1]	0.371
Fluoroscopy exposure (min, median) [range]	N/A	5.3 [2.5-32.8]	N/A
Fluoroscopy before sample collection (min, median) [range]	N/A	1.4 [0.2-20.2]	N/A
Complications, n (%)	1/65 (1.5)	3/64 (4.7)	0.304

There was one complication in the VBNA group [i.e., hyperventilation (*n* = 1)], and 3 complications in the XRFA group [i.e., hemorrhage (*n* = 2) and pneumonia (n = 1)]. The incidence of complications did not differ between the 2 groups, and no severe adverse effects were observed in either group.

## Discussion

Guided-bronchoscopy technologies including endoscopic virtual navigation technology have advanced and contributed to improvement of the diagnostic yield [13]. Here, we performed a multicenter non-inferiority study to demonstrate that the combination of VBN and EBUS-GS enables the bronchoscopic diagnosis of peripheral pulmonary lesions without X-ray fluoroscopy in a procedural series (guidance of the bronchoscope, confirmation of the lesion using EBUS/GS, and biopsy) similar to EMN, but this was not demonstrated. To our knowledge, this is the first randomized controlled study on VBN without X-ray fluoroscopy.

On EBUS/GS, arrival of the biopsy instruments at the lesion can be confirmed and biopsy can be repeatedly applied [8]. Yoshikawa et al. performed a feasibility study of EBUS/GS-guided transbronchial biopsy (TBB) of 123 peripheral lesions without X-ray fluoroscopy although VBN was not concomitantly used, in which the diagnosis yield of 2-cm or larger lesions, bronchus sign-positive lesions, and solid lesions were high: 75.8, 79.2, and 67.0%, respectively, showing its usefulness [12]. Since the target was set at large lesions with a 3-cm or larger size in the present study, all lesions were bronchus sign-positive excluding 3 cases in the XRFA group. However, the diagnostic rate using EBUS/GS without X-ray fluoroscopy was only 76.9% even though VBN was concomitantly used, and non-inferiority to X-ray fluoroscopy-assisted EBUS/GS could not be demonstrated, clarifying that VBN cannot substitute for X-ray fluoroscopy in the bronchoscopic diagnosis of pulmonary peripheral lesions when used together with EBUS/GS.

Ishida et al. demonstrated that VBN increased the diagnostic yield of 3-cm or smaller peripheral lesions by transbronchial lung biopsy using EBUS/GS and X-ray fluoroscopy in a multicenter randomized controlled study [25]. VBN shortened the total examination time, especially the time to sample collection. The duration of X-ray fluoroscopy was also shortened, although the difference was not significant. Asano et al. performed a multicenter randomized controlled study on transbronchial lung biopsy of 3-cm or smaller peripheral lesions using an ultrathin bronchoscope, in which VBN increased the rate of biopsy instrument arrival at the lesion [26]. In the present study, agreement between the virtual bronchoscopic images and actual bronchi was 93.8%, being high, and the endoscopically inserted bronchial generation was larger and enabled deep and accurate insertion in the VBNA group, similarly to the situation in the 2 studies described above [25, 26]. Normally, VBN presents a route to the lesion on virtual images up to the bronchus peripheral from the 4-mm bronchoscope insertion range (median: 6th generation of the bronchus [26]). In the VBNA group, the bronchoscope was actively inserted toward a peripheral site along the route presented on virtual images as much as possible, which may have resulted in the more peripheral bronchoscope insertion range compared with that in the XRFA group. The rate of visualizing lesions by EBUS, i.e., the rate of the biopsy instrument reaching the lesion, was 95.4% (62/65) in the VBNA group and 96.9% (62/64) in the XRFA group, being high in both groups with no significant difference. It was clarified that a bronchoscope and biopsy instruments can be correctly guided to the lesion using VBN without X-ray fluoroscopy, i.e., VBN can substitute for X-ray fluoroscopy with regard to guidance. The total examination time was also shorter in the VBNA group in the present study, although the difference was not significant.

On the other hand, in the study performed by Ishida et al., the diagnostic yield was 80.4% even when the lesions were 3 cm or smaller in the VBN + EBUS + X-ray fluoroscopy group, but the diagnostic yield was low, 76.9%, in the present study due to the non-use of X-ray fluoroscopy even though the lesions were 3 cm or larger. Normally, X-ray fluoroscopy is used to guide a bronchoscope and biopsy instruments to the lesion before sample collection, and confirm opening and closing of the forceps during sample collection and the absence of deviation of the biopsy instrument from the lesion. The low diagnostic yield may have been due to the absence of X-ray fluoroscopy during sample collection. In EBUS/GS, open forceps do not come out of the guide sheath so that opening or closing of the forceps can be identified without X-ray fluoroscopy. When forceps are not sufficiently open, a sufficient amount of specimen cannot be collected from the lesion, but the degree of opening of the forceps cannot be confirmed without X-ray fluoroscopy. Moreover, the guide sheath tip may deviate from the lesion as biopsy is repeated but this cannot be determined when X-ray fluoroscopy is not used. It is complicated and unrealistic to confirm the presence of the EBUS probe and guide sheath tip in the lesion in each biopsy on EBUS. The diagnostic yield of visualized lesions by EBUS to which the biopsy instrument may have reached was 80.6% in the VBNA group, being lower than that (88.7%) in the XRFA group, and this may have been due to insufficient opening of the forceps and deviation of the guide sheath tip from the lesion during biopsy. The necessity of X-ray fluoroscopy for EMN during sample collection has not been described [16]. In EMN, the position of the guide sheath tip is unlikely to deviate because it is curved, and the size of usable forceps is larger. In the present EBUS/GS, the guide sheath tip was straight and thinner than that of EMN. Moreover, the forceps used were also thin, and so the forceps have a weaker opening force than the normal ones and may not open in the lesion. To sufficiently open the forceps, it was necessary to rapidly push and pull the forceps tip little by little at the site while opening them in many cases, which may have been a cause of deviation of the guide sheath from the lesion. The diagnostic yield of brush biopsy was also lower in the VBNA group, and this may have been due to deviation of the guide sheath from the involved bronchus caused by applying brush biopsy after forceps biopsy. Improvement of the guide sheath and forceps may be necessary. These problems may be more important when the lesion is smaller. Yoshikawa et al. stated that the advantages of X-ray fluoroscopy are as follows: The position of the lesion can be seen from a three-dimensional perspective, curettage is applicable, and respiratory movement of the lesion can be identified [12].

VBN is useful to guide a bronchoscope to peripheral lesions, but EBUS is necessary to confirm their arrival at the lesion in real-time. Inversely, the arrival of the biopsy instrument at the lesion can be confirmed by EBUS/GS, but guidance to the lesion using EBUS/GS alone is difficult. The combination of these is complementary and effective, but accurate and efficient sample collection from the lesion without X-ray fluoroscopy is difficult. Although it is not possible to omit X-ray fluoroscopy from the procedure of bronchoscopic diagnosis of peripheral lesions using VBN, unlike that using EMN, the use of VBN allows operators to shorten the duration of X-ray fluoroscopy as much as possible. It is important to collect specimens from the lesions efficiently within a short time by appropriately combining these 3 modalities and achieve a high diagnostic yield. Since the target was limited to 3-cm or larger lesions in order to investigate the possibility of EBUS without X-ray fluoroscopy in this study, it is necessary to investigate whether a bronchoscope and biopsy instruments can be similarly guided to small lesions using VBN without X-ray fluoroscopy.

## Conclusions

On EBUS/GS, a bronchoscope and biopsy instruments may be guided to lesions using VBN without X-ray fluoroscopy, but X-ray fluoroscopy is necessary because the accuracy and efficiency of sample collection from lesions decreases without its use, and so VBN does not substitute for X-ray fluoroscopy in the bronchoscopic diagnosis of peripheral lesions.

## Abbreviations

EBUS: Endobronchial ultrasonography
EBUS/GS: Endobronchial ultrasonography with a guide sheath
EMN: Electromagnetic navigation
VBN: Virtual bronchoscopic navigation
VBNA: Virtual bronchoscopic navigation-assisted
XRF: X-ray fluoroscopy
XRFA: X-ray fluoroscopy-assisted
